# The Application of the Haddon Matrix to Public Health Readiness and Response Planning

**DOI:** 10.1289/ehp.7491

**Published:** 2005-02-02

**Authors:** Daniel J. Barnett, Ran D. Balicer, David Blodgett, Ayanna L. Fews, Cindy L. Parker, Jonathan M. Links

**Affiliations:** Johns Hopkins Center for Public Health Preparedness, Johns Hopkins Bloomberg School of Public Health, Baltimore, Maryland, USA

**Keywords:** dirty bombs, emergency, Haddon matrix, injury prevention, preparedness, public health, readiness, response, SARS, terrorism

## Abstract

State and local health departments continue to face unprecedented challenges in preparing for, recognizing, and responding to threats to the public’s health. The attacks of 11 September 2001 and the ensuing anthrax mailings of 2001 highlighted the public health readiness and response hurdles posed by intentionally caused injury and illness. At the same time, recent natural disasters have highlighted the need for comparable public health readiness and response capabilities. Public health readiness and response activities can be conceptualized similarly for intentional attacks, natural disasters, and human-caused accidents. Consistent with this view, the federal government has adopted the all-hazards response model as its fundamental paradigm. Adoption of this paradigm provides powerful improvements in efficiency and efficacy, because it reduces the need to create a complex family of situation-specific preparedness and response activities. However, in practice, public health preparedness requires additional models and tools to provide a framework to better understand and prioritize emergency readiness and response needs, as well as to facilitate solutions; this is particularly true at the local health department level. Here, we propose to extend the use of the Haddon matrix—a conceptual model used for more than two decades in injury prevention and response strategies—for this purpose.

## Hypothetical Cases

### SARS Preparedness and Response

It was an unseasonably warm Friday morning on 12 March 2004 in Anytown, Maryland. Since 1 March 2004, the Department of Homeland Security had raised the U.S. terror alert level to code orange (high) based on fresh intelligence reports from interviews with Al Qaeda detainees at Guantanamo Bay.

The Baltimore Orioles were in the process of gearing up for another season. On Monday, 8 March 2004, 75 diehard baseball fans returned to Dulles Airport on Orioles Airways Flight 000, after watching the Orioles play a series of spring training exhibition games in Florida over the weekend.

One of the passengers on this Orioles Airways Flight 000 was Mr. Smith, an Anytown, Maryland, businessman who had traveled to Taipei, Taiwan, for meetings during the week of 1 March 2004. He had taken a direct flight to Taipei from Dulles Airport on Monday, 1 March, with a stopover that day in Munich, Germany; he had flown back to Dulles on Thursday, 4 March, also with a stopover in Munich. Upon returning to Dulles, he spent the night at a hotel in McLean, Virginia. He flew the next morning, 5 March, from Dulles to Fort Lauderdale, Florida, on Orioles Airways Flight 007 to watch his beloved Orioles play a weekend’s worth of spring training games, before returning to Dulles on the 8 March Orioles Airways Flight 000.

Early on the morning of 8 March, before boarding Flight 000, Mr. Smith developed a sudden fever and dry cough, along with chills and muscle aches. Despite these symptoms, after the flight he still managed to drive from Dulles Airport to Anytown, Maryland. Within 2 hr of arriving at his apartment to his wife and two children in Anytown, Mr. Smith’s condition rapidly deteriorated, and he began to have difficulty breathing. His wife drove him to General Hospital emergency department in Anytown.

Mr. Smith was admitted to the intensive care unit at General Hospital on 8 March, with a suspected clinical diagnosis of severe acute respiratory syndrome (SARS).

Three days later (11 March), doctors at one hospital in Washington, DC, one hospital in Baltimore, and General Hospital in Anytown admitted three patients each (total = 9 patients) with histories of acute onset of high fever (> 38°C) and dry cough followed by shortness of breath.

Upon taking a detailed travel history of these patients, physicians determined that seven of these nine patients (including the three new patients presenting to General Hospital in Anytown) had taken Orioles Airways Flight 000 on 8 March 2004. Two others had recently traveled to the United States from Guangdong Province, China. These developments were reported on a 24-hr cable media outlet before local, state, and federal public health officials had a chance to generate a formal press release.

Meanwhile, at General Hospital in Anytown, the condition of Mr. Smith steadily worsened despite aggressive treatment efforts, and he died of respiratory failure on the afternoon of 11 March.

By 2000 hr on 11 March, local, national, and international media outlets had converged upon Anytown, with a sea of television trucks and satellite equipment gathered outside General Hospital. The 911 system became flooded with calls from anxious citizens throughout Anywhere County, and cell phone networks were quickly overwhelmed by call volume. The mayor of Anytown, Maryland, and the local county health commissioner prepared to deliver a joint press conference with the state health commissioner at 2030 hr, followed by an address by the president to the nation on these developments at 2100 hr.

By 13 March 2004, a total of 90 cases of SARS were confirmed in Maryland, Pennsylvania, northern Virginia, and the District of Columbia. Twenty of these patients had died thus far from respiratory failure. The news of these deaths brought added fear to the region and the nation. Schools had been closed and unnecessary gatherings canceled in Anytown and the rest of the affected region for the past 2 days.

Epidemiologic workup by the Centers for Disease Control and Prevention (CDC) in conjunction with state and local health departments revealed that most cases in this SARS outbreak were traceable to Mr. Smith, the Anytown businessman who had been exposed to SARS while on business in Taipei and who subsequently exposed fellow passengers on Orioles Airways Flight 000 because of a faulty on-plane ventilation system. The remaining cases were traced to the two travelers to Baltimore who came from Guangdong Province in China.

#### Questions.

What are the hospital infection control issues associated with a SARS outbreak, and what are the most effective approaches to address these issues? What type of advance planning strategy could a local public health department use to identify the contributing factors to this public health emergency? What approaches could a local public health department use to deliver comprehensive public health prevention, intervention, and risk communication measures before, during, and after such an outbreak?

### “Dirty Bomb” Preparedness and Response

It was late in the afternoon on a typically warm, humid, sunny 4 July afternoon in Anytown, Maryland. Thousands were gathered at the Anywhere County fairgrounds in Anytown in preparation for that evening’s upcoming parade and celebration, and the crowds were currently enjoying an outdoor concert and other festivities. Police estimated the afternoon’s crowd at the fairgrounds at approximately 10,000.

There was a breeze blowing westward at 10 miles/hr, cooling the fairground crowd slightly and making them a little more comfortable. Tens of thousands more were en route to Anytown for the evening’s celebration via the major highways, including I-95, I-495, and I-270. There was heavy freeway congestion at this hour outside downtown Anytown. Warnings from the Department of Homeland Security had been issued for vigilance during the 4 July holiday weekend, but the nature of this terrorist threat had been nonspecific, and the nation had been at a U.S. terror alert level of code yellow on this 4 July holiday.

It was estimated that 7,500 of the 10,000 people at the fairgrounds this afternoon were attending the concert. About 30 min into the show, a man driving a white van on Any Parkway suddenly stopped at the main entrance to the fairgrounds, about 50 yd from the concert venue. Ten seconds later the van exploded in a massive fireball, the blast hurling fiery shrapnel into the crowd.

The explosion killed 300 people instantly and injured 2,000 more in the adjacent crowd, and the blast could be heard over a 5-mile radius. Smoke emanating from the resulting fire was visible to motorists on the congested freeways and roads leading to the fairgrounds.

Within moments of the blast, thousands of people began fleeing from the fairgrounds. Motorists hearing the blast and seeing the smoke from area freeways and roads began to use their cell phones simultaneously by the thousands. Cellular phone systems rapidly became flooded.

On Monday, 8 July, an Associated Press wire bulletin surfaced that three moisture density gauges—each containing 10 mCi cesium-137—were first reported missing that morning from a construction site on Mary-land’s Eastern Shore. The site manager said the gauges were last seen on 1 July, the day before the construction crew left the site for the extended holiday weekend.

Given this new information, public safety authorities had a high index of suspicion that this terrorist blast may have been caused by a “dirty bomb” containing the cesium-137 from the Eastern Shore construction site. Environmental sampling revealed elevated radiation levels at the site of the explosion, consistent with this hypothesis.

In the several weeks after the attacks, emergency rooms noted a surge in patients coming in for anxiety-related symptoms. Area pharmacies were flooded with prescriptions for anxiolytic and antidepressant medications. Community mental health services were being strained as Anytown citizens attempted to come to grips with the horror of this terrorist attack. Many residents of Anytown stated they would never return to the city again because they believed the area would never be adequately decontaminated.

#### Questions.

What are the potential environmental impacts of a dirty bomb? What can be done to prepare for and respond to such impacts? How would local, state, and federal public health and partner emergency response agencies work together in this scenario? What steps would be taken to distinguish a dirty bomb vs. from another type of explosion? What steps would be taken to evacuate, contain, and decontaminate the affected area? Would evacuation involve all of Anywhere County? Who would take the lead in communicating timely, accurate information to the public on radiation terror before, during, and after this event? What would the crisis- and consequence-phase mental health service responses be to an attack on Anytown by a “dirty bomb”? What steps, if any, could have prevented this attack from occurring or could have reduced the number of deaths and injuries?

## Discussion

### The Haddon matrix.

The field of injury prevention has long provided solution-oriented models for understanding threats to the public’s health. Industry and public health officials alike have applied these models to reduce morbidity and mortality from a variety of injury types. The Haddon matrix, developed by William Haddon, has been used for more than two decades in injury prevention research and intervention. The Haddon matrix is a grid with four columns and three rows. The rows represent different phases of an injury (pre-event, event, and postevent), and the columns represent different influencing factors (host, agent/vehicle, physical environment, social environment). Table 1 illustrates a basic application of the Haddon matrix to pedestrian traffic safety.

The host column represents the person or persons at risk of injury. The agent of injury impacts the host through a vehicle (inanimate object) or vector (person or other animal/organism). Physical environment refers to the actual setting where the injury occurs. Sociocultural and legal norms of a community constitute the social environment. The phases of an event are depicted on the matrix as a continuum beginning before the event (preevent), the event itself (event phase), and sequelae of the event (postevent phase).

The terminology used for the factors of the matrix can be adapted for different contexts; for example, “agent” may be more appropriate than “vector” in certain cases, and “organizational culture” might be used in addition to or instead of “social environment” (Tables 2–4) when focusing on an institutional context.

Through its phase-factor approach, the Haddon matrix meshes concepts of primary, secondary, and tertiary prevention with the concept of the host/agent/environmental interface as a target for delivering public health interventions (Runyan 1998). Each cell of the matrix represents a distinct locus for identifying strategies to prevent, respond to, or mitigate injuries or other public health challenges (Runyan 1998). By dissecting a problem into its dimensions of time and contributing factors, the Haddon matrix can be applied as a practical, user-friendly interdisciplinary brainstorming and planning tool to help understand, prepare for, and respond to a broad range of public health emergencies (Runyan 2003).

#### The Haddon matrix and new readiness challenges for public health.

As an integral component of homeland security in the post–11 September environment, the public health infrastructure faces new and significant challenges of recognizing and responding to a broad range of intentional and naturally occurring large-scale threats. Furthermore, since the anthrax attacks of 2001, the concept of public health emergency preparedness in the United States has evolved and expanded from a bioterrorism focus to an all-hazards readiness and response model. The all-hazards approach means that the infrastructure and skill sets used to prepare for and respond to a bioterrorism event can also be applied to a wide spectrum of current and emerging natural and intentional threats to the public’s health, ranging from an infectious disease outbreak to a weather-related disaster.

Effective public health emergency preparedness and response requires appropriate preevent, event (crisis phase), and postevent (consequence phase) activities. In the context of emergency readiness, preevent activities include risk assessment, risk communication, and primary prevention efforts (e.g., preevent vaccination). Event-phase public health activities involve crisis risk communication and community-based medical interventions such as postexposure prophylaxis and treatment, crisis mental health counseling, and isolation/quarantine measures. Postevent activities involve consequence-phase disaster mitigation and treatment of longer-term physical and mental health sequelae, along with ongoing risk communication and recovery efforts.

Table 2 presents a conceptual overview of public health emergency preparedness and response activities and competencies and how they might be illustrated using the Haddon matrix. Items with asterisks on Table 2 are CDC-adopted emergency preparedness competencies for all public health workers developed by the Columbia University School of Nursing Center for Health Policy (2002). This high-level view of the issues faced by those preparing for emergencies demonstrates the multidimensional flexibility of the Haddon matrix.

Each phase of a public health emergency presents a unique set of demands on health departments in their readiness and response efforts. Allocating resources for these phases is a significant challenge in the face of competing public health priorities and resource demands. These preevent/event/postevent phase challenges and the organizational flexibility requirements of an all-hazards response model can quickly become overwhelming for public health departments.

By breaking a larger problem into smaller, more manageable components, the Haddon matrix provides a practical, efficient decision-making and planning tool that health department leaders can use to better understand current and emerging threats, perform vulnerability assessments, prioritize and allocate readiness and response resources, and maintain institutional agility in responding to an array of public health emergencies.

Health department leaders can use the Haddon matrix as a planning instrument to dissect the required preparedness and response requirements for any public health emergency scenario, and then strategize to meet these requirements using a “divide and conquer” approach. Once the Haddon matrix has been filled in for a given type of emergency, the cells of the completed matrix comprise specific preevent, event, and postevent task-oriented items that leaders can assign to appropriate staff to optimize their agency’s readiness and response. Some of these items within the completed Haddon matrix may be more responsive than others to public health prevention and intervention, or may represent more pressing needs for a given community; this allows health department leaders to prioritize these assigned tasks based on the health department’s unique demands and resources.

The Haddon matrix can also serve as a helpful after-action evaluation tool to assess a health department’s performance in achieving the goals of a preparedness exercise, or in responding effectively to a real-life event. In this context, the tasks within each cell become items for performance evaluation that can contribute to an effective, comprehensive after-action report.

A view of readiness challenges through the lens of the Haddon matrix also promotes efficient use of public health resources, because the matrix can reveal strategies that allow multiple issues to be addressed by one solution. For example, the logistics of trying to anticipate every possible source of attack or emergency are staggering and impractical. The establishment of an effective incident command system and flexible emergency operations plan within a health department facilitates a more effective response regardless of the emergency. Through the use of the Haddon matrix, it becomes much more likely that public health departments will be able to maximize their readiness efforts, because policies and procedures that are identified as clearly beneficial in multiple scenarios can be developed ahead of less generalizable efforts.

The Haddon matrix also promotes efficient resource allocation by focusing on appropriate phase responses. Because the matrix requires the user to follow issues across all of the phases of an event, problems that seem insurmountable during one phase might have ready solutions in a different phase. For example, the logistics of adequately sheltering a population upon the release of an infectious disease become much more manageable with a “preevent” educated population that understands the concepts of sheltering in place, emergency supply kits, and resources for additional trustworthy information.

The model shows considerable flexibility as a tool to address threats—both intentional and unintentional—that face public health departments in their efforts to enhance public health readiness and response. From SARS to dirty bombs, the Haddon matrix reveals itself as a useful public health readiness tool for tackling difficult public health emergencies.

#### SARS preparedness and response: a Haddon matrix analysis.

SARS is an example of a naturally occurring public health epidemic that can be better understood and addressed via the Haddon matrix. From diagnosis, to treatment, to infection control, to risk communication, SARS is an infectious disease that exacts significant stress on multiple facets of the public health infrastructure (Affonso et al. 2004; Gostin et al. 2003).

A myriad of public health response issues surround a SARS outbreak. Table 3 shows an example of the Haddon matrix as applied to one such issue: SARS hospital infection control. This SARS model of the Haddon matrix views infectious disease as a form of injury affecting the population on a broad scale. The model allows its users to better understand the multidimensional nature of the epidemic and to identify targets for prevention, mitigation, and intervention. By identifying targeted points of intervention (noted with asterisks in Table 3), we can discover potential measures to successfully mitigate the public health threat before, during, and after a SARS event.

Table 3 illustrates some of the hospital infection control factors that should be considered in the event of an emerging infectious disease outbreak such as SARS (Loutfy et al. 2004; Svoboda et al. 2004). Lessons on public health readiness are often learned painfully after large crises, as was the case during the SARS outbreak of 2003 (Campbell 2004; Hearne et al. 2004). Using the Haddon matrix before an event occurs allows us to consider the interplay of variables that might otherwise have been missed (and were missed during the actual events associated with the SARS outbreak). For example, in the pre-event phase under physical environment, the Haddon matrix reveals the importance of addressing the need for adequate personal protective equipment; this may seem obvious enough in hindsight, but this issue received insufficient attention before the SARS outbreaks in 2003 (Campbell 2004; Reznikovich and Balicer 2004).

Equally important, the model is flexible enough to allow for big picture analysis of a situation, or a more focused analysis of the smallest units of study, including individuals. As a tool to understand, prepare for, and respond to SARS, the Haddon matrix thus reveals itself as a highly adaptable model.

#### “Dirty bomb” preparedness and response: a Haddon matrix analysis.

From a public health emergency readiness standpoint, the Haddon matrix’s adaptability also extends to environmental impacts of nonbiologic origin. Radiation terror preparedness, for example, is a significant challenge in the emerging all-hazards public health readiness framework, because the physical and mental health impacts of radiation terror on an affected area can be profound and long lasting.

Radiologic dispersal devices (“dirty bombs”) are examples of radiation terror that present a challenge for homeland security because of their simplicity and relative ease of acquisition. Dirty bombs are conventional explosives bundled with ionized radioactive sources, and remain a front-line terrorism preparedness concern in the post-11 September era (Zimmerman and Loeb 2004).

Applying the Haddon matrix to the threat of a dirty bomb illustrates the value of this injury prevention model as a public health readiness and response tool, even when focusing exclusively on environmental issues. Table 4 shows how the Haddon matrix can be applied to address environmental health issues related to dirty bombs. Although the human, agent, physical, and social factors are numerous, a closer look reveals a more specific set of points for targeting environmental assessment and intervention (Table 4).

Like the Haddon matrix for SARS in Table 3, the Haddon matrix for dirty bombs in Table 4 reveals the host, social environmental/organizational culture, and selected physical environmental dimensions as major points of impact for public health assessment and intervention (noted with asterisks). Hazardous materials (Hazmat) and other first-responder agency personnel would comprise the front lines at the scene of a dirty bomb event, rather than health department workers. Nonetheless, a comparison between the dirty bomb and SARS Haddon matrix examples shows marked similarities in the importance of risk communication, mental health support, resource use, surge capacity, and effective surveillance as points of public health impact, consistent with an all-hazards readiness and response framework.

Table 4 reveals that from an environmental perspective, modifiable public health “impact” opportunities for dirty bomb preparedness and response involve mainly organizational culture/social environment factors, as well as a few host and physical environment factors. The legal and regulatory aspects of environmental remediation after a dirty bomb are critical public health issues with significant economic implications (Elcock et al. 2004); these are also reflected in Table 4 as “impact” opportunities on the Haddon matrix.

Collectively, these modifiable host, physical environment, and social environment/organizational culture factors represent targets for streamlining readiness and response activities; addressing the safety, risk perception, and mental health needs of first responders and Hazmat personnel; and managing the financial resource and response issues of a dirty bomb—all of which are critical pieces in dealing with the environmental impacts of a dirty bomb.

## Conclusion

The applied examples of SARS and dirty bombs illustrate the utility and flexibility of the Haddon matrix as a tool for understanding, preparing for, and reacting to a spectrum of intentional and naturally occurring public health threats.

Following the principle that “all disasters are local,” the Haddon matrix can provide a tool for public health agencies to address specific gaps and requirements that must be filled to meet their communities’ unique readiness needs. Additionally, the Haddon matrix can serve as a helpful model for disaster preparedness and response in a variety of contexts, from public health readiness policy development to local public health practice emergency response planning.

As an effective creative brainstorming and planning tool, it is ideally suited to facilitate tabletop preparedness exercises at health departments in cooperation with partner first-response agencies. It can assist in needs assessment efforts for public health agencies and their stakeholders. It also can serve as a valuable classroom aid in teaching public health readiness concepts at the secondary and graduate school levels, helping future public health leaders to develop critical problem-solving skills needed to tackle difficult readiness challenges.

These examples and their potential applications highlight five essential features of the Haddon matrix as a tool for public health emergency readiness and response. First, the Haddon matrix provides a framework for understanding a terrorism incident in a temporal context, including its preevent, event (crisis), and postevent (consequence) phases. Second, it can effectively dissect these temporal phases of a public health event into their contributing factors. Third, it can aid in a public health agency’s vulnerability assessment of its preparedness and response capacities. Fourth, it can provide health departments with a useful framework for developing these capacities to deliver a prioritized, targeted approach to the public health dimensions of terrorism prevention and response. Fifth, it is a sufficiently flexible analytic tool to aid health departments in addressing virtually any type of intentional or naturally occurring public health emergency.

The dissection of SARS and dirty bombs by the Haddon matrix reveals how widely disparate public health challenges can be tackled by a user-friendly and efficient injury prevention conceptual model. A renewed look at the Haddon matrix thus shows this tool to be a vital link between public health preparedness and injury prevention science.

## Figures and Tables

**Table 1:** The Haddon matrix and pedestrian injury from automobiles.

	Influencing factors			
Phase	Host	Agent/vehicle	Physical environment	Social environment
Preevent	Intoxicated driver	Speeding automobile	Poor street lighting	Unenforced speed limit laws
	Fatigued driver	Worn tires	Slick pavement	Inadequate investment in crosswalks
	Pedestrian crossing street	Worn brakes	Potholes Inadequate signage Nighttime	
	Intoxicated pedestrian Elderly pedestrian			
	Pedestrian with osteoporosis	Momentum of automobile		
Event	Pedestrian wearing headphones		Hospitals nearby with specialty in trauma care	Good samaritan laws
	Hearing-impaired pedestrian	Impact of automobile with pedestrian		
	Part of pedestrian’s body struck by vehicle	Portion of vehicle impacting pedestrian	Part of body impacting ground	
Postevent	Ability of victim to recover Postinjury care received	Severity of physical injuries	Rehabilitation facility	Health insurance
		Severity of postevent psychological impact		
				Access to rehabilitation services Family and social support
	Psychological coping of victim in aftermath of event			

**Table 2:** The Haddon matrix and public health emergency readiness and response—a conceptual overview.

	Influencing factors			
Phase	Host	Agent/vector	Physical environment	Social environment/organizational culture
Preevent	Risk assessment	Properties of biologic, chemical radiologic, or other agents	Existing clinical infrastructure Vulnerability of food and water supplies	Need for culture of readiness among public health and other first responders
	Preevent risk communication	Capacity of agent as WMD	Transportation infrastructure	Knowing one’s functional role(s) in emergency response*
	Preevent surveillance	Potential for re-engineering of agent to produce unexpected health effects		Demonstrating use of communication equipment*
	Primary prevention (e.g., preevent vaccination)		Proximity of community to chemical and radiation facilities	Knowing one’s communication role(s) in emergency response*
	Preparedness training for public health responders			Identifying key system resources for referring matters that exceed one’s personal knowledge and expertise*
	Interagency first response planning			Participation in readiness exercises and drills Baseline community trust in public health and other response agencies Public acceptance of preevent risk communication Culturally based preevent risk perception Public awareness of large-scale threats Demographics of community
Event	Crisis risk communication	Disease or injury caused by agent	Emergency response clinic setup and operations	Community responses to crisis risk communication
	Decontamination and treatment	Response of the agent to decontamination and treatment efforts	Emergency access to medical supplies (e.g., Strategic National Stockpile)	Community adherence to public health guidance during event
	Sheltering	Potential for agent detection	Clinical surge capacity	Culturally based crisis-phase risk perception
	Postexposure prophylaxis	Psychosocial impact of agent during event	Shelter availability	Access of community to crisis response clinics
	Crisis-phase mental health response Crisis-phase interagency first response collaboration Epidemiological workup (including forensic epidemiology as applicable) Evacuation	Acute health effects of agent	Emergency accessibility of transportation	
Postevent	Consequence-phase risk communication	Long-term psychosocial impact of agent	Application of lessons learned to better safeguard vulnerable infrastructure	Community responses to postevent risk communication
	Application of lessons learned to improve response systems Consequence-phase mental health response	Response of agent to mitigation and cleanup efforts		Willingness of public health responders to embrace lessons learned
				Postevent community trust in public health and other response agencies
	Postevent health surveillance Mitigation and cleanup After action assessment and follow-up			Culturally based consequence-phase risk perception

WMD, weapons of mass destruction.

* Potential targets for public health intervention.

**Table 3:** The Haddon matrix and SARS hospital infection control.

	Influencing factors			
Phase	Host	Agent/vector	Physical environment	Social environment/organizational culture
Preevent	Preevent training of staff in outbreak infection control practices*	Level of contagiousness	Availability of PPE*	Preevent employee awareness of daily infection control practices*
	Case mix of patients in the hospital	Incubation period	Availability of predesignated outbreak infection control checklists and forms*	Organizational culture of staff adherence to hospital directives and protocols*
	Surveillance for SARS within hospital by health care providers*	Subclinical infection	Hospital infection control infrastructure (e.g. negative pressure rooms)*	Cultural competency of preevent risk communication to hospital staff*
	Preevent public health risk communication*	Level of contagiousness	Laboratory facilities*	Budget (preparedness resource allocation)*
		Lethality	Plans for increased surge capacity*	
		Potential modes of transmission	Proximity of hospital to international airports and borders*	
Event	Mental health support for hospital staff during event*	Mode(s) of dissemination of virus during actual outbreak	Hospital surge capacity	Hospital staff’s trust in administrators’ crisis management performance
	Staff adherence to hospital infection control protocols		Availability of designated SARS hospitals in vicinity	Budget (response resource utilization)
	Isolation and quarantine implementation		Communication network systems capacity	Incident command system put into action*
	Risk communication during event to staff and patients*		Crisis-designated incident command system for hospital infection control	Media accuracy and bias toward health care providers
			Efficiency of medication and equipment delivery (e.g., Strategic National Stockpile)*	Culturally and scientifically appropriate/consistent SARS messages to hospital staff and patients* Moral support to affected health care community* Patient and family compliance with hospital infection control protocols
Postevent	Postevent risk communication	Persistence of agent in environment	Postevent decontamination options for affected facility	Cultural competency of postevent messages*
	Postmortem management		Restoration of Strategic National Stockpile medication and equipment*	Governmental financial support of affected hospitals*
	Psychology of postevent reactions			Ongoing mental health support and followup*
	Postevent surveillance			Economic impact on affected community

PPE, personal protective equipment.

* Potential targets for public health intervention.

**Table 4:** The Haddon matrix and environmental impact of dirty bombs.

	Influencing factors			
Phase	Host	Agent/vehicle	Physical environment	Social environment/organizational culture
Preevent	Malicious intent of terrorist	Sources of ionizing radiation	Fresh water	First responders’ preevent risk perception of radiation terror*
	Access of terrorist to explosives and radiation	Types of ionizing radiation (electromagnetic vs. particulate)	Power supply	Cultural competency of preevent risk communication messages to first responders*
	Level of Hazmat teams’ preparedness and training	Properties of ionizing radiation (e.g., half-life, carcinogenicity)	Security of industrial/medical facilities where radiation is stored*	Awareness of first responders to public health threat of radiation terror*
	Preevent surveillance of environmental radiation*		Availability of PPE for Hazmat teams	Existing laws and regulations on radiologic cleanup*
			Availability of decontamination equipment for Hazmat teams	Budget (preparedness resource allocation)*
			Availability of communication equipment* Availability of radiation detection equipment for non-Hazmat first responders Proximity of community to radiologic hazards Climate Geography	Insurance
Event	Malicious execution of terrorist act	Mode of radioactive material dispersion: air, water, soil, or food	Weather conditions during event	Cultural competency of public health messages for first responders*
	Implementation of detection and decontamination efforts		Proper functioning of decontamination equipment	Incident command system put into action*
	Intra-agency and interagency communications and collaboration*		Communication systems surge capacity*	Budget (response resource utilization)*
		Transportation systems	Executive orders by elected officials and community compliance	
			Time, distance, and shielding of affected communities	
Postevent	Physical and psychological impacts on Hazmat personnel and first responders	Persistence of agent in environment	Weather (e.g., wind direction, temperature)	Cultural competency of postevent public health messages*
	Postevent environmental surveillance of radiation*	Postevent control options based on agent and mode of dispersion (cleanup, disposal)		Economic impact on affected community
	Postevent risk communication*			Environmental remediation and regulation* Postevent media coverage

PPE, personal protective equipment.

* Potential targets for public health intervention.
